# Effects of Equine-Assisted Therapy: A Systematic Review and Meta-Analysis

**DOI:** 10.3390/jcm14113731

**Published:** 2025-05-26

**Authors:** Alexandra N. Stergiou, Avraam Ploumis, Spyridon Kamtsios, Georgios Markozannes, Pineio Christodoulou, Dimitrios N. Varvarousis

**Affiliations:** 1 Department of Primary Education, University of Ioannina, 45110 Ioannina, Greece; 2 Division of Physical Medicine and Rehabilitation, School of Medicine, University of Ioannina, 45110 Ioannina, Greece; aploumis@uoi.gr; 3 Department of Psychology, University of Ioannina, 45110 Ioannina, Greece; spiroskam@gmail.com; 4 Department of Hygiene and Epidemiology, School of Medicine, University of Ioannina, 45110 Ioannina, Greece; 5 Department of Education, School of Education, University of Nicosia, 2417 Nicosia, Cyprus; xripinio@gmail.com; 6 Department of Anatomy, School of Medicine, University of Ioannina, 45110 Ioannina, Greece; dimvarvar@gmail.com

**Keywords:** equine-assisted therapy, cerebral palsy, multiple sclerosis, elderly, stroke, physical disabilities

## Abstract

**Objectives:** Different types of exercises that aim in the development of balance, motor function, and gait are necessary for patients with motor disorders. Equine-assisted therapy could play an important role in the rehabilitation of these participants. **Methods:** The purpose of this study was to examine the effects that equine-assisted therapy can exert on balance, motor function, spasticity, posture and gait, as well as quality of life on individuals with motor disorders. Clinical trials, published up to 20 April 2022, comparing equine-assisted therapy with conventional rehabilitation were systematically searched. Two independent reviewers performed data extraction and assessed the quality of studies using the Downs and Black quality assessment tool. **Results:** Out of 27 studies that satisfied the inclusion criteria for systematic review, 15 included appropriate data for further comparative meta-analysis. Statistically significant differences were found in Dimension E (walking, running, jumping) of Gross Motor Function Measure in children with CP (0.009) and in Time Up and Go in Elderly and post-stroke participants (*p* = 0.006). Specifically, children with CP improved in walking, running, and jumping, as well as improved mobility in the elderly. The systematic review showed that the intervention had positive results, as well as in other domains, even though these were not statistically significant. **Conclusions:** Equine-assisted therapy is beneficial for individuals with impairments in balance, gross motor function, gait, spasticity, and coordination.

## 1. Introduction

Equine-assisted therapy (EAT) provides many benefits to individuals with neuromotor, developmental, and physical disabilities, including the improvement of functional abilities and balance. Also, it may delay progression of some disorders, ultimately leading to reduced morbidity and reduced premature mortality [1,2,3]. The psychological benefits deriving from EAT in these patients are also important [4]. Individuals with motor dysfunction have abnormal gait patterns due to abnormal muscle tone, reduced control of their muscles, incoordination, asymmetry between agonist and antagonist muscles, and poor equilibrium reflexes [4].

EAT improves posture, balance, mobility, walking energy expenditure, function, and sensory abilities. As horseback riding rhythmically moves the rider’s body in a manner similar to a human gait, riders with physical disabilities often show improvement in flexibility, balance, muscle strength, coordination, in the range of motion of joints, in weight shifts [1], in the stability of the hip, the pelvis and the trunk [5], as well as in reduction of the oscillation due to the effort to remain stable on the horseback [6]. In addition to the therapeutic benefits, EAT also provides recreational opportunities for such individuals to enjoy the outdoors.

Literature reviews and meta-analysis studies [7,8,9,10] from the international literature reveal the efficacy of EAT across populations with neuromotor, developmental, and physical disabilities, using outcome measures including GMFM, balance tests, fatigue scales, and quality of life assessments. These studies consistently found short-term improvements in mobility, posture, balance, fatigue reduction, and quality of life, particularly through EAT. However, significant research gaps remain due to a lack of standardized intervention protocols and outcome measures, limited data on long-term effects and underlying mechanisms, inconsistent prioritization of motor outcomes, scarce cost-effectiveness analyses, and a need for broader population diversity in study samples.

This study presents a systematic review and meta-analysis to evaluate the effects of EAT on balance, motor function, spasticity, posture, gait, quality of life, and overall well-being in individuals with neuromotor, developmental, and physical disabilities. Drawing on evidence from the international literature, the review incorporates recent randomized controlled trials and other relevant studies, enabling a more comprehensive synthesis of the available data. By providing a quantitative summary of current findings, this review aims to enhance the existing body of knowledge and contribute to a clearer understanding of EAT’s therapeutic benefits. The research question poses the following question: In individuals with neurological, developmental, or physical disabilities, how does equine-assisted therapy, compared to standard therapy or no intervention, affect motor function, balance, spasticity, posture, gait, quality of life, and overall well-being?

## 2. Materials and Methods

### 2.1. Data Sources

This study was designed and reported in accordance with the PRISMA (Preferred Reporting Items for Systematic Reviews and Meta-Analyses) statement [11]. This study conforms to all PRISMA guidelines and reports the required information accordingly [12]. The statement was registered at the International Prospective Register of Systematic Reviews (PROSPERO) (CRD42021228253).

A comprehensive literature search was conducted across the following databases: MEDLINE, Scopus, CINAHL, EMBASE, SportDiscus, ISI Web of Science, Cochrane Database of Systematic Reviews, Google Scholar, Cochrane Central Register of Controlled Trials, DARE, PEDro, and Dissertation Abstracts. The search utilized relevant keywords, titles and, where possible, abstracts to identify studies on EAT. The literature research was conducted until the 20 of April 2022. The search algorithm used was “therapeutic riding” OR “therapeutic horse riding” OR “therapeutic horseback riding” OR “horse riding” OR “horseback riding” OR hippotherapy OR “equine-assisted therapy” OR “ equine-assisted activities” OR “equine-assisted movement therapy” OR “equine therapy” OR “equine movement therapy” OR “developmental riding therapy” OR “riding for the disabled”. The search was limited to English language articles.

### 2.2. Eligibility Criteria

The studies included in this review consist of published trials (in English) involving children, adults, and older adults diagnosed with conditions associated with motor function impairments. The focus was on evaluating the effects of EAT on balance, postural control, gait, spasticity, and overall quality of life. A key inclusion criterion was that the outcomes of EAT were assessed quantitatively.

#### Inclusion and Exclusion Criteria

Only studies with a control/comparison group have been included. The studies excluded included (i) studies not providing data on baseline score or end-point outcome, (ii) single subject studies, (iii) studies providing only qualitative data, (iv) case series studies, and (v) studies that used a mechanical horse. Exclusion criteria were set for co-interventions such as medication or surgery that might have influenced the outcome. To ensure the reliability and objectivity of the citation selection process, all potentially relevant studies were independently reviewed by two investigators.

### 2.3. Data Extraction

The following data were extracted from each eligible study: first author, year of publication, country, total sample size, sample size per disorder type, participant characteristics and descriptive statistics (e.g., mean age), details of interventions by disorder type, intervention duration, outcomes assessed, follow-up period, measurement tools used to evaluate intervention efficacy, and baseline and endpoint values. Additionally, any adjuvant interventions administered were also recorded. Data extraction was conducted by the lead author and verified by a co-author to ensure accuracy. One author was contacted by email requesting additional information regarding outcomes not reported in her manuscripts and responses were received.

### 2.4. Methodological Quality Assessment of Research Articles

The methodological quality of each study was independently assessed by two co-authors, with any discrepancies resolved by a third reviewer. Evaluation was conducted using the Downs and Black quality assessment tool, which consists of 27 items grouped into four categories: reporting (10 items), external validity (3 items), internal validity (7 items related to bias and 6 items related to confounding), and power (1 item). Twenty-five items were scored as follows: “yes” = 1 point, “no” = 0 points, and “unable to determine” = 0 points. Item 5 was scored as “yes” = 2 points, “partially” = 1 point, and “no” = 0 points. The final item, item 27, was scored on a scale from 0 to 5 points. The total Downs and Black score for each study thus ranged from 0 to 32 points [13]. The kappa statistic was used in order to determine the interrater reliability, a measurement of the extent to which data collectors (raters) assign the same score to the same variables [14,15].

### 2.5. Statistical Analysis

A meta-analysis was conducted to evaluate the mean differences between the intervention and control groups for the following outcomes: the Berg Balance Scale (BBS) [16] or Pediatric Balance Scale (PBS) [17], the Gross Motor Function Measure (GMFM) [18] total score and Dimensions A-E, the Time up and Go (TUG) [19], the Tinetti Performance Oriented Mobility Assessment (POMA) [20] and Child Health Questionnaire (CHQ-28)—physical domain [21]. Random effects meta-analysis models were used. The meta-analysis was based on the inverse variance method for weighting and the Dersimonian and Laird estimator [22]. Cochran’s Q test statistic [23] was employed to assess heterogeneity across the studies. The degree of heterogeneity was quantified using the I^2^ statistic, which ranges from 0% to 100% and cut-off values of 25%, 50%, and 75% indicate low, moderate, and high degrees of heterogeneity, respectively. Both randomized and non-randomized studies were included in the meta-analysis. A subgroup analysis using results only from randomized studies was also performed. The analysis was conducted with the use of Stata version 13 (College Station, TX, USA).

## 3. Results

**Figure 1:**
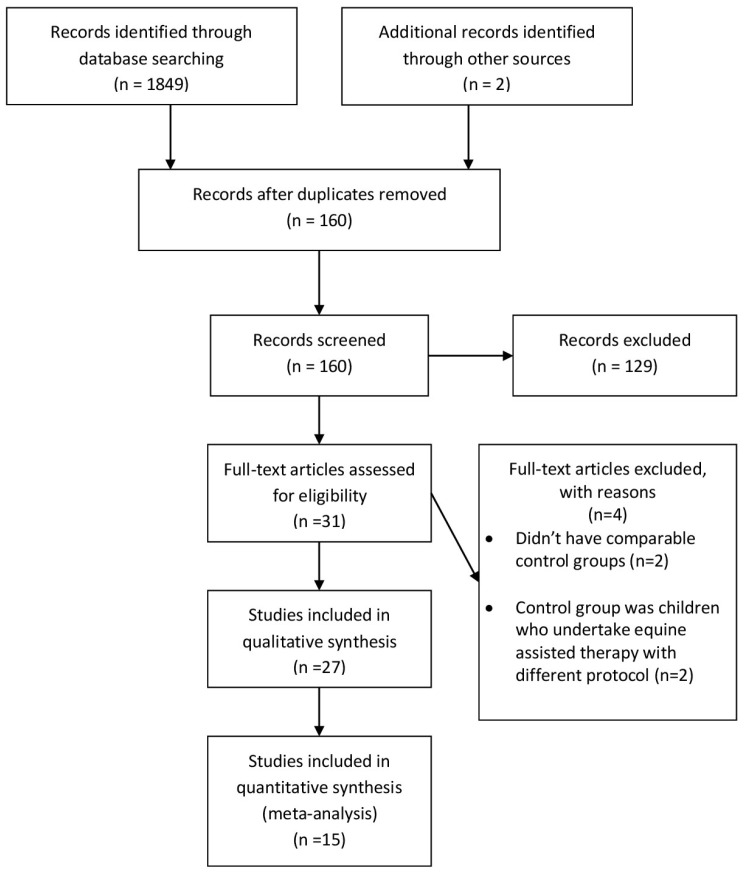
Prisma 2009 Flow Diagram [24].

The literature review and the application of the eligibility and inclusion/exclusion criteria led to the detailed assessment of 27 studies. (Table 1, Figure 1).

**Table 1:** Profiles of included studies in the meta-analysis.

Studies	Randomized Total (IG/CG)	Age (Years) Mean ± SD	Gender N (%)	Classification	Intervention/Control Details	Study Duration	Intervention/ Control	Outcomes
**Randomized CP studies**				**GMFCS**				
Lucena-Anton (2018) [25]	44 (22/22)	IG: 9.5 ± 2.74 CG: 8227 ± 2.42	IG: 13M 9F CG: 15 M 7F	IV–V	IG: 45 min/week CG: 2 sessions/week	12 weeks	EAT/Conventional therapy	MAS
Matusiak-Wieczorek (2020) [26]	45 (15/15/15)	IG: 7.93 ± 2.6 IG: 7.6 ± 1.84 CG: 8.13 ± 2.56	IG: 9M 6F IG: 8M 7F CG: 8M 7F	I–II	IG: 30 min/twice a week IG: 30 min/week CG: -	12 weeks	EAT/EAT/Not received EAT	SAS
Mutoh (2019) [27]	24 (12/12)	IG: 8 ± 3 CG: 9 ± 3	IG: 5M (42) CG: 6M (50) IG: 7F (58) CG: 6F (50)	II–III	IG: 30 min/week CG:weekly recreation program Follow-up: 3 month	1 year	EAT/Recreation program	5 MWT WHOQOL-BREF GMFM-66
Deutz (2018) [28]	73 (35/38)	IG: 9.29 ± 3.7 CG: 8.87 ± 2.9	IG: 23M 12F CG: 21M 17F	II–IV	IG: once–twice/week CG: once–twice/week	32–36 weeks	35 early treatment/ 38 late treatment	GMFM-66 CHQ-28 KIDSCREEN-27 parental versions
Kwon (2015) [29]	91 (45/46)	IG: 5.7 ± 1.9 CG: 5.9 ± 1.8	IG: 20M (44) CG: 29M (63) IG: 25F (56) CG: 17F (37)	I–IV	IG: 30 min/twice a week CG: 30 min/twice a week	8 weeks	EAT/Home-based aerobic exercise (walking or cycling)	GMFM-88 PBS
Davis et al. (2009) [30]	99 (50/49)	IG: 7.7 ± 2.4 CG: 8.2 ± 2.5	IG: 26M (52) CG: 27M (55.1) IG: 24 (48) CG: 22 (44.9)	I–III	IG: 30–40 min/week CG: normal daily/weekly routines	10 weeks	EAT/Usual activities	Cerebral Palsy Quality of Life Questionnaire for Children GMFM-66 KIDSCREEN CHQ-28
Kang (2012) [31]	43 (14/15/14)	IG: 8.2 ± 1.1 PTG: 8.2 ± 1.1 CG: 7.8 ± 1.5	IG: 7M 7F PTG: 8M 7F CG: 7M 7F	Not Reported	IG: 30 min/semi-weekly PTG: 30 min/semi-weekly CG: -	8 weeks	EAT/Physiotherapy/No treatment	Force plate (PMD Multifunction Force Measuring Plate; Zebris, Gemany, 2004)
Benda et al. (2003) [32]	15 (7/8)	4–12 years	Not Reported	Not Reported	IG: 8 min CG: 8 min	1 session	EAT/Stationary Barel	electromyography
**Non-Randomized CP studies**								
Kwon (2011) [33]	32 (16/16)	IG: 6.4 ± 1.7 CG: 6.1 ± 1.7	IG: 11M 5F CG: 10M 6F	I–II	IG: 30 min/twice a week CG: 30 min/twice a week	8 weeks	EAT/Neurodevelopmental therapy	Gait Analysis (Vicon 612 Motion Analysis System) GMFM-88 PBS
Park (2014) [34]	55 (34/21)	IG: 6.68 ± 2.64 CG: 7.76 ± 3.67	IG: 15M 19F CG: 10M 11F	I–IV	IG: 45 min/twice a week CG: 30 min/week	8 weeks	EAT/Outpatient physical and occupational therapy	GMFM-66 GMFM-88 PEDI-FSS
Matusiak-Wieczorek (2016) [35]	39 (19/20)	IG: 8.42 ± 2.24 CG: 8.3 ± 2.62	IG: 10M 9F CG: 11M 9F	I–II	IG: 30 min/week CG: -	12 weeks	EAT/Usual activities of daily living and attended different forms of rehabilitation	SAS
Alemdaroglu (2016) [36]	16 (9/7)	Total: 7.5 ± 1.7	9 M (56) 7 F (44)	I–V	IG: 2 times 30 min/week CG: 5 days/week	5 weeks	EAT/Conventional rehabilitation program	(MFRT) Goniometric measurement of hip abduction AS
MacKinnon (1995) [37]	19 (10/9)	Total: 6.5 ± 6.5	IG: 3M 7F CG: 6M 3F	mild and moderate	IG: 60 min/once a week CG: -	26 weeks	EAT/waiting list	GMFM Bertoti scale PDMS Bruininks–Oseretsky test of motor proficiency Vineland Adaptive Behaviour Scale Harters Self-Perception Scale CBC
Baik (2014) [38]	16 (8/8)	IG: 12.2 ± 3.6 CG: 8.12 ± 2.58	Not Reported	Not Reported	IG: 60 min/twice a week CG: not reported	12 weeks	EAT/Rehabilitation	MAS Passive goniometer measure ROM
**Randomized Elderly studies**								
White-Lewis (2019) [39]	20 (10/10)	IG: 61.9 ± 6.05 CG: 65.80 ± 7.42	IG: 4M (40) CG: 0M (0) IG: 6F (60) CG: 10F (100)		IG: 60 min/week CG: 60 min/week	6 weeks	EAT/Exercice Education	hand-held goniometer measure ROM VAS Likert Scale AIMS 2
Diniz (2020) [40]	30 (15/15)	IG: 66.07 ± 5.8 CG: 68.47 ± 5.85	IG: 4M 11F CG: 2M 13F		IG: 30 min/week CG: -	10 weeks	EAT/Daily activities without physiotherapy	BBS TUG FRT Sit-and-Reach Test
Araujo (2013) [41]	28 (12/16)	IG: 65.59 ± 6.5 CG: 65.81 ± 6.6	IG: 4M 8F CG: 2M 14F		IG: 30 min/twice a week CG: -	8 weeks	ΕAΤ/Daily activities without physiotherapy	TUG 30 CST BBS
Kim (2014) [42]	22 (11/11)	IG: 70.3 ± 3.4 CG: 68.5 ± 3.2	IG: 5M 6F CG: 7M 4F		IG: 20 min/three times/week CG: 20 min/three times/week	8 weeks	ΕAΤ/Treadmill	BPM Force platform (5.3, SMS Health care Inc., UK)
**Non Randomized Elderly studies**								
Homnick (2015) [43]	15 (9/6)	IG: 70.1 CG: 69.3	IG: 2M 7F CG: 3M 3F		IG: 45 min/week CG: -	10 weeks	ΕAΤ/Usual activities	BBS FABS
Araujo (2011) [44]	17 (7/10)	60–84	IG: 2M 5F CG: 0M 10F		IG: 30 min/twice a week CG: -	8 weeks	ΕAΤ/Controls	A Stabilometer (AMTI AccuSway Plus using Balance Clinic software) TUG
**Randomized MS studies**				**Pattern of MS**				
Vermöhlen (2018) [45]	67 (30/37)	IG: 50 CG: 51	IG: 3M (10) CG: 10M (27) IG: 27F (90) CG: 27F (73)	Reported 2 RR	IG: once/week CG: -	12 weeks	EAT/Continued previous therapy	BBS FSS MSQoL-54 VAS NRS
Moraes (2020) [46]	33 (17/16)	IG: 45.5 ± 9.7 CG: 44.8 ± 8.8	IG: 1M 16F CG: 1M 15F	RR	IG: 30 min/twice a week CG: -	8 weeks	EAT/ Maintained therapeutic routine	T25FW 6 MWT
**Non Randomized MS studies**								
Silkwood-Sherer (2007) [47]	15 (9/6)	IG: 42.4 ± 14.2 CG: 47.7 ± 9.3	IG: 4M 5F CG: 2M 4F	8 RR 4 PR	IG: 40 min/week CG: not reported	14 weeks	EAT/Controls	BBS POMA
Munoz-Lasa (2011) [48]	27 (12/15)	IG: 45.8 CG: 46.2	IG: 5M 7F CG: 6M 9F	14 RR 9 SP 4 PP	IG: 30–40 min/week CG: 30–40 min/week	10 weeks- 4 weeks resting period-10 weeks	EAT/Physiotherapy	POMA Barthel Index
**Randomized Stroke studies**								
Bunketorp-Kall (2017) [49]	82 (41/41)	IG: 62.6 ± 6.5 CG: 63.7 ± 6.7	IG: 24M (58.5) CG: 22M (53.7) IG: 17F (41.5) CG: 19F (46.3)		IG: 2 sessions/week CG: - Follow-up: 6 months	12 weeks	EAT/Controls	SIS (version 2.0) TUG BBS BDL-BS Grippit Barrow Neurological Institute screen for higher cerebral functions Letter–number sequencing test
Bunketorp-Kall (2019) [50]	82 (41/41)	IG: 62.6 ± 6.5 CG: 63.7 ± 6.7	IG: 24M (58.5) CG: 22M (53.7) IG: 17F (41.5) CG: 19F (46.3).		IG: 2 sessions/week CG: - Follow-up: 6 months	12 weeks	EAT/Controls	10 MWT 6 MWT M-MAS
**Non Randomized Stroke studies**								
Beinotti (2010) [51]	20 (10/10)	IG: 59 CG: 52	IG: 8M 2F CG: 6M 4F		IG: hippotherapy once/week + conventional therapy twice/week CG: 3 times/week Follow-up: 6 months	16 weeks	EAT/Conventional treatment	Functional Ambulation Category Scale Fugl–Meyer Scale BBS Cadence

AIMS 2: Arthritis Impact Measurement Scale 2; AS: Ashworth Scale; BBS: Berg Balance Scale; BDL-BS: Bäckstrand, Dahlberg and Liljenäs Balance Scale; CBC: Child Behavior Checklist; CG: Control group; CHQ: Child Health Questionnaire; EAT: Equine-Assisted Therapy; FABS: Fullerton Advanced Balance Scale; FRT: Functional Reach Test; FSS: Fatigue Severity Scale; GMFM: Gross Motor Function Measure; GMFCS: Gross Motor Function Classification System. IG: Intervention Group; MAS: Modified Ashworth Scale; MFRT: Modified functional reach test; MSQoL-54: Multiple Sclerosis Quality of Life-54; NRS: Numeric Rating Scale; PBS: Pediatric Balance Scale; PDMS: Peabody developmental motor scale; PEDI-FSS: Pediatric Evaluation of Disability Inventory: Functional Skills Scale; POMA: Tinetti Performance Oriented Mobility Assessment; PR: Primary progressive; PTG: Physical therapy group; ROM: Range of Motion; RR: relapsing remitting; SAS: Sitting Assessment Scale; SD: standard deviation; SIS: Stroke Impact Scale; SP: secondary progressive; TUG: Time up and Go; T25FW: 25-foot walk test; VAS: Visual Analogue Scale; WHOQOL-BREF: World Health Organization Quality of Life; 5 MWT: 5 min walk test; 6 MWT: 6 min walk test; 10 mWT: 10 m walk test; 30 CST: 30 s Chair Stand Test.

### 3.1. Quality Assessment

Agreement between the two raters was 97.2%, and the interrater reliability kappa statistic [52] was equivalent to 0.93 (standard error, 0.027), indicating substantial agreement. The mean (standard deviation—SD) Quality Index Score for randomized controlled trials was 24.8 (5.1) and for non-randomized studies 19.7 (2.6).

**Figure 2:**
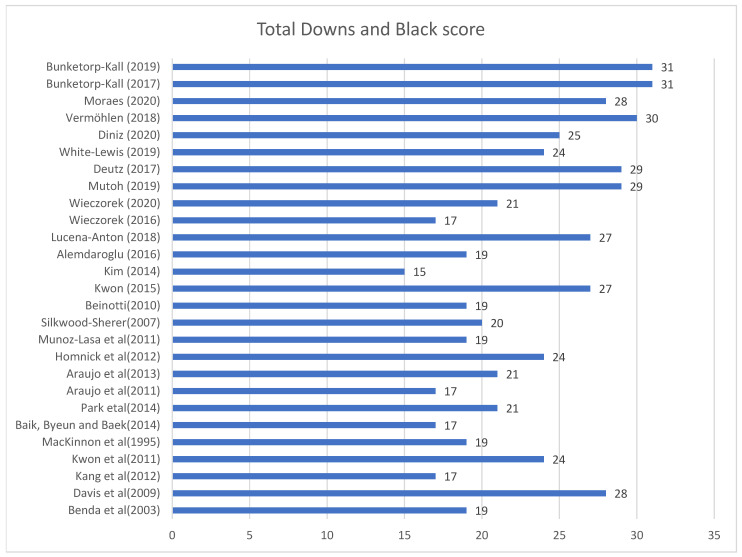
Downs and Black diagram for selected studies.

The lowest score was noted for Kim et al. (2014) [42], with a score of 15, and the highest was noted for Bunketorp-Kall et al. (2017) [49] and (2019) [50], with a score of 31 (Figure 2). From the 27 items of the Downs and Black checklist, the items that had the highest score were 1 (hypothesis/objectives clearly described), 13 (staff, places, facilities where patients were treated is representative of the treatment the majority of patients receive), 14 (blind subjects), 16 (if results based on “data dredging” was this made clear?), 17 (in trials and cohort studies, do the analyses adjust for different lengths of follow-up of patients, or in case-control studies, is the time period between the intervention and outcome the same for cases and controls?), 19 (compliance with intervention reliable), 20 (main outcome measures accurate, valid, and reliable) and 21 (patients in different groups recruited from the same population). On the other hand, item 24 (was the randomized intervention assignment concealed from both patients and health care staff until recruitment was complete and irrevocable?) had the lowest score, in which only two studies pointed as yes (they included this criterion).

#### 3.1.1. Cerebral Palsy

Fourteen studies evaluated the effects of EAT in children with Cerebral Palsy (CP), involving a total of 611 participants. Of these, 311 children were assigned to the intervention group, while 300 were included in the control group.

Six studies [25,28,29,33,34,37] found statistically significant results between two groups in motor function, three [36] in standing balance, three [26,31,35] in [29,33] sitting balance, two [27,33] in gait parameters, three in the reduction of spasticity [25,36,38], one [32] in the symmetry of muscle activity, one [38] in the joint range of motion, and finally four studies [27,28,30,37] showed improvements in psychosocial domains and quality of life. The common outcome measures were the GMFM [18] and PBS [17].

#### 3.1.2. Elderly Individuals

Six randomized clinical trials investigated the effectiveness of EAT in improving mobility among the elderly. A total of 140 participants were included, with 68 assigned to the intervention group and 72 to the control group.

Four of the above studies [41,42,43,44] showed significant improvements in balance, three [40,41,44] in functional mobility, one [40] in flexibility, and finally one study that investigated the reduction of pain and quality of life in elder individuals with arthritis. The study of White-Lewis 2019 [39] was included in the systematic review because it is considered that there is a limitation both in the CNS and musculoskeletal system. The common outcome was the TUG [19] and the BBS [16].

#### 3.1.3. Multiple Sclerosis (MS)

Four studies examined the effects of EAT on individuals with MS, involving a total of 142 participants. Of these, 68 were assigned to the intervention group, while the remaining participants formed the control group. Among the 141 individuals for whom diagnostic details were available, 55 had relapsing-remitting MS, 8 had primary progressive MS, and 9 had secondary progressive MS. In the study by Silkwood-Sherer and Warmbter (2007) [47], the MS type was unspecified for 3 participants, while the study by Vermohlen et al. (2018) [45] did not report the MS subtype for 67 participants.

Three [45,47,48] studies showed significant improvement in balance, two [46,48] in gait parameters, and one [45] in fatigue, spasticity, and quality of life. The common outcome measures were the POMA [20], the BBS, and the gait speed (m/s).

#### 3.1.4. Stroke

Three randomized clinical trials have evaluated the effects of EAT on individuals recovering from stroke. The total number of participants was 102, where 51 participants made up the intervention group and 51 the control group. It was noticed that in both Bunketorp-Kall et al.’s (2017) [49] and (2019) [50] studies, the same number of participants were used, but different outcome measures were studied.

Two of the above studies [49,51] showed improvements in balance, three [49,50,51] in functional mobility, and one [49] in participants’ perception of stroke recovery.

### 3.2. Meta-Analysis

#### 3.2.1. Berg Balance Scale (BBS) and Pediatric Balance Scale (PBS)

Nine studies examining the effects of EAT, using the BBS and PBS as outcome measures, met the inclusion criteria for the meta-analysis. These included three studies on the elderly, two on individuals with MS, two on stroke patients, and two on children with CP. A meta-analysis was performed for each of the four groups of participants (Table 2). A total of 187 participants were included in the intervention group and 193 in the control group. The PBS is an adapted scale of BBS for children.

For the elderly individuals group, the analysis revealed a mean difference (improvement) of 0.11 points in the BBS score; however, this difference was not statistically significant (*p* = 0.889). The estimated 95% confidence interval (C.I.) for the pooled mean difference was (−1.46 to 1.68). This analysis presented significant heterogeneity (I^2^ = 77.4%; *p* = 0.012).

For the other groups, among participants with multiple sclerosis, the analysis indicated an improvement, with a mean difference of 2.48 points on the BBS; however, this difference was not statistically significant (*p* = 0.255). The estimated 95% C.I. for the pooled mean difference was (−1.79 to 6.75). Similarly, in participants with stroke, a mean difference (improvement) of 0.99 points on the BBS was observed, but this too was not statistically significant (*p* = 0.492), with a 95% C.I. for the pooled mean difference was (−1.84 to 3.83). No significant heterogeneity was observed for participants with multiple sclerosis and stroke, with I^2^ = 0.0% (*p* = 0.559 and 0.623, respectively).

In participants with cerebral palsy, the analysis showed a mean improvement of 3.21 points on the PBS; however, this difference was not statistically significant (*p* = 0.21). The estimated 95% C.I. for the pooled mean difference was (−1.82 to 8.24), and I^2^ was 0.0% (*p* = 0.629), showing no heterogeneity.

#### 3.2.2. Time up and Go (TUG)

**Figure 3:**
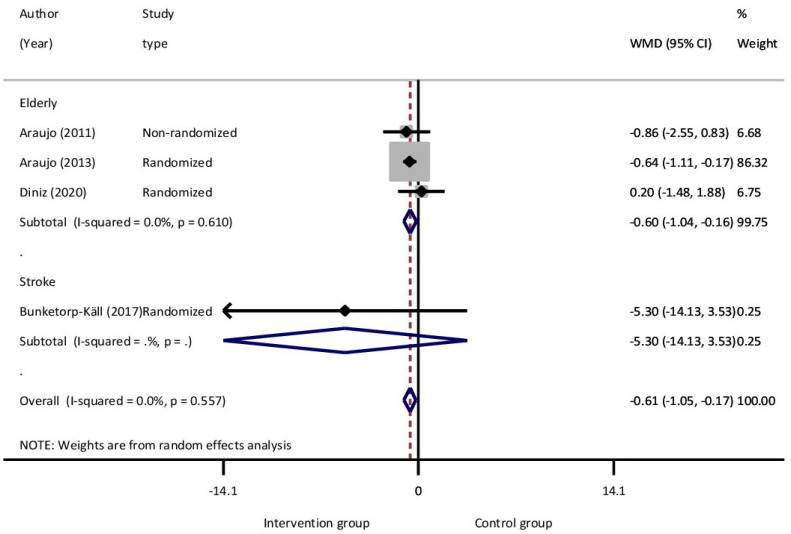
Subgroup synthesis forest plot. Meta-analysis Time up and Go (TUG).

Five studies investigated the effects of EAT using TUG, and they met the inclusion criteria for the meta-analysis. Three of these studies involved elderly participants, while one focused on stroke individuals. The number of participants included was 75 in the intervention group and 82 in the control. A meta-analysis was performed for each of the two groups of participants (elderly and post stroke patients) and also as a whole, as the participants of stroke had similar ages with those of the elderly studies. The analysis for these groups found statistically significant improvement, with a mean difference of 0.60 and 0.61 s, respectively (*p* = 0.007 and 0.006). The estimated 95% C.I. for the pooled mean difference for the elderly group was −1.04 to −0.16 and overall −1.05 to −0.17. For the elderly group and also for the common elderly and stroke group, there was no heterogeneity, with I^2^ = 0.0% (*p* = 0.610 and 0.557, respectively) (Table 2 and Figure 3).

#### 3.2.3. Gross Motor Function Measure (GMFM)

**Figure 4:**
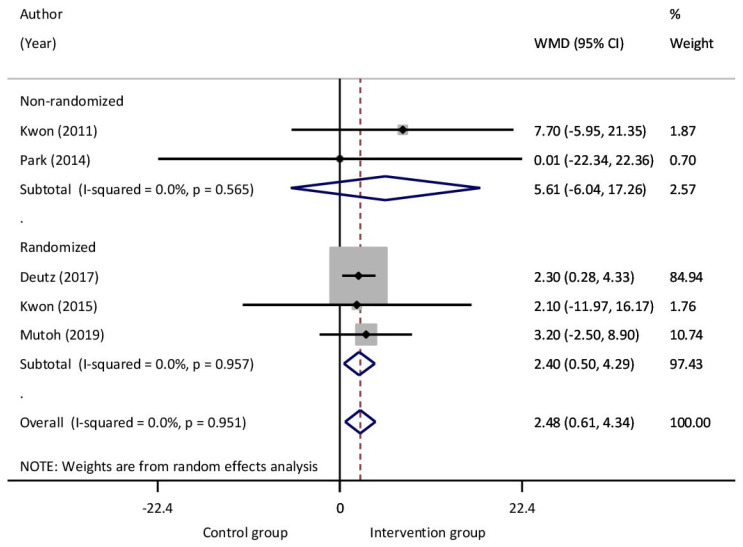
Subgroup synthesis forest plot. Meta-analysis Gross Motor Function Measure (GMFM) Dimension E.

Reported results from six studies were used to examine the effect of EAT on children with CP, which included 162 patients in the intervention and 159 patients in the control group. The analysis of Dimension E (walking, running, jumping) of the GMFM reached statistically significant improvement, with a mean difference of 2.48 points (*p* = 0.009). No statistically significant results were found in GMFM-88, GMFM-66, or Dimensions A, B, C, and D between the intervention and non-intervention group, although there was an improvement in mean differences (2.92, 0.67, 0.26, 9.79, 8.47, and 0.11 points, respectively). No significant heterogeneity was observed in GMFM-88, GMFM-66, or its dimensions, except Dimensions B and C, in which they observed significant heterogeneity (I^2^ = 91.1% and 70.5 with *p* = 0.001 and 0.066, respectively) (Table 2, Figure 4).

#### 3.2.4. Tinetti Performance Oriented Mobility Assessment (POMA)

In terms of POMA, two studies including participants with multiple sclerosis were used to examine the effects of EAT. There were 21 participants in the intervention group, and 20 were the controls. The analysis showed that there was an improvement, with a mean difference of 2.32 points, but this was not statistically significant (*p* = 0.149). The estimated 95% C.I. for the pooled mean difference was −0.83 to 5.46. The I^2^ statistic was 0.0% (*p* = 0.585), showing no heterogeneity (Table 2).

#### 3.2.5. Child Health Questionnaire (CHQ)28—Physical Domain

Reported results from two studies were used to examine the quality of life in the physical domain of children with CP. A total of 55 children were included in the intervention and 64 in the control group. The analysis showed that there was an improvement, with a mean difference of 3.82 points, but this was not statistically significant (*p* = 0.175). The estimated 95% C.I. for the pooled mean difference was −1.70 to 9.34. The I^2^ statistic was 0.0% (*p* = 0.367), showing no heterogeneity (Table 2).

## 4. Discussion

This systematic review and meta-analysis aimed to evaluate the effects of EAT on balance, motor function, spasticity, posture, gait, and quality of life in individuals with neuromotor, developmental, and physical disabilities. By synthesizing findings from diverse populations, including children with cerebral palsy (CP), elderly individuals, patients with multiple sclerosis (MS), and stroke survivors, this study provides an updated, comprehensive assessment of the therapeutic benefits of EAT.

Τhis meta-analysis found statistically significant differences in GMFM Dimension E (walking, running, jumping) in children with CP and in TUG in elderly and post-stroke participants. The systematic review also showed that the intervention had positive results in other domains, even though these were not statistically significant.

Generally, in individualized studies, it was observed that EAT had a significantly positive impact on adults with MS and stroke, on children with CP, as well as elderly individuals with multiple health problems and disabilities. More specifically, it was observed that EAT significantly improved the posture, motor function, balance, gait, pelvic movement, muscle symmetry, psychosocial parameters, and overall quality of life.

It was possible to conduct a meta-analysis separately for each dimension of the GMFM, and a statistically significant result was observed for GMFM Dimension E (walking, running, jumping). Although not statistically significant, positive effects of EAT on the other individual dimensions (A—lying and rolling, B—sitting, C—crawling and kneeling, and D—standing) and on the total score were also observed based on the point estimates of the meta-analyses. Statistically significant results also emerged for the TUG in stroke and elderly individuals. The same statistically significant results remained also when only randomized trials were analyzed. Specifically, for the TUG, the results did not change when stroke individuals were assessed together with elderly individuals. No statistically significant results were observed in the Child Health Questionnaire (CHQ) and Tinetti Performance Oriented Mobility Assessment (POMA) for children with CP and adults with MS, respectively. Finally, this meta-analysis, similar to a previous one [2], which included the BBS/PBS, did not demonstrate statistically significant benefits of EAT compared to other forms of therapy. While three trials investigated spasticity, they provided incomparable data (different spasticity scales or different muscle group), so it was not possible to include them in the meta-analysis.

### 4.1. Impact of EAT on Specific Populations

#### 4.1.1. Cerebral Palsy

Among children with CP, EAT demonstrated notable improvements in motor function, balance, and spasticity reduction, supported by specific findings from the GMFM and PBS. Analysis of GMFM Dimension E (walking, running, and jumping) revealed a statistically significant mean improvement of 2.48 points (*p* = 0.009), emphasizing the therapy’s positive impact on dynamic motor activities. While the PBS analysis showed a mean difference of 3.21 points, suggesting potential clinical relevance, this result was not statistically significant (*p* = 0.21; 95% C.I.; −1.82 to 8.24). The lack of heterogeneity (I^2^ = 0.0%; *p* = 0.629) enhances confidence in these findings. Additionally, EAT’s benefits extended to psychosocial domains and quality of life, highlighting its holistic therapeutic potential for children with CP. These results provide quantitative evidence supporting EAT as an effective intervention for enhancing motor skills and overall well-being in this population.

Previous systematic reviews and meta-analyses [53,54,55] have shown improvement in gross motor function, muscle strength, balance, gait, and quality of life in children with CP through interventions such as aerobic exercises, resistance training, treadmill training, neurodevelopmental therapy, and balance training. In our systematic review, it is observed that EAT improves muscle activity symmetry [32], sitting balance [26,31], walking speed, stride length, and pelvic kinematics in GMFM (Dimension E: walking, running, and jumping), in balance [27,29,33], in Skill A (grasping) of Fine Motor Control [37], in spasticity [25,36,38], and in quality of life [27,28]. Furthermore, only Dimension E of GMFM showed statistically significant improvement between the intervention and control groups. One previous meta-analysis of EAT [56] shows the same results with the current study. They found significant improvements in GMFM-E and not in the other GMFM dimensions, gait parameters, or quality of life. Another meta-analysis [7] found a statistically significant improvement in lower-limb muscle spasticity in children with CP but with a high level of heterogeneity. This study did not perform a meta-analysis using the Modified Ashworth Scale due to an insufficient number of available studies.

#### 4.1.2. Elderly Individuals

EAT demonstrated significant improvements in balance, functional mobility, and flexibility among elderly participants, as evidenced by the results of the TUG test. Among elderly individuals, a statistically significant mean reduction in TUG time of 0.60 s was observed (*p* = 0.007; 95% C.I.; −1.04 to −0.16), indicating enhanced mobility and reduced fall risk. Similarly, a pooled analysis that included elderly participants and individuals with stroke—who had comparable ages—showed a mean reduction of 0.61 s (*p* = 0.006; 95% C.I.; −1.05 to −0.17). Notably, there was no evidence of heterogeneity (I^2^ = 0.0%; *p* = 0.610 for elderly group; *p* = 0.557 for pooled group), further strengthening the findings. These results underscore the potential of EAT as a therapeutic intervention for mitigating age-related declines in motor function and promoting active aging. Additionally, the recreational and engaging nature of EAT may contribute to improved mental well-being and adherence to therapy within this demographic.

In elderly individuals, exercise and various combinations of interventions were associated with lower risk of injurious falls [57]. Systematic reviews and meta-analyses [58,59] have shown that exercise interventions improve physical function, as indicated by increased muscle strength, gait speed, mobility, balance and physical performance, and cardiorespiratory and functional fitness, as well as improvements in daily living activities in this population. TUG and BBS are common instruments that measure mobility and balance, respectively, but only TUG has shown statistically significant results in the meta-analysis. A systematic review of Lea Badin [60] found similar results with the present study regarding effectiveness in the physical domain, including balance, gait, and strength. Concerning physiological effects, equine-assisted intervention has similar effects to those observed during sports, which means that changes happened in hormone levels and in brain activity. It increases levels of serotonin (a hormone responsible for well-being) and decreases the secretion of cortisol (a hormone responsible for stress).

Regarding the psychological domain, the same study [60] refers to improvements in quality of life and well-being. In contrast, some studies show that exercise has no significant impact on elderly individuals’ quality of life [57,58]. However, our systematic review did not find any studies that fulfill the inclusion criteria regarding the physiological domain, quality of life, or well-being in elderly populations.

#### 4.1.3. Multiple Sclerosis

Participants with MS experienced improvements in balance, gait parameters, and reductions in fatigue and spasticity. While the POMA and Berg Balance Scale (BBS) analyses revealed positive trends, these results were not statistically significant, possibly due to small sample sizes and heterogeneity in MS progression. The overall benefits suggest that EAT may offer a complementary approach to conventional therapies for MS, addressing both physical impairments and psychosocial challenges.

Resent systematic reviews and meta-analyses [61] showed that physical exercise reduces fatigue in patients with MS. Also, an increase in cardiorespiratory fitness, muscle strength and endurance, and in the ability to perform daily tasks was observed. Another study [62] demonstrated significant results in muscle strength with progressive resistance training. Previous research also revealed that exercise had beneficial effects on emotional and physical function in persons with depressive symptomatology [63]. The current systematic review agrees with the results of other systematic reviews conducted for individuals with MS after performing physical exercise on horseback [64]. More specifically, it was noticed that EAT improved balance [45,48], reduced stride time and ground reaction forces [48], and generally improved quality of life [45]. However, these results were not supported by the results of the current meta-analysis. On the contrary, a meta-analysis by Suarez-Iglesias et al. (2021) [10] found significant results in static balance, fatigue, and quality of life for people with MS after EAT. They did not observe significant changes in gait and dynamic balance.

#### 4.1.4. Stroke

Stroke survivors participating in EAT programs showed notable improvements in functional mobility and balance. While the mean difference in BBS scores was positive, it did not reach statistical significance, likely due to the limited number of studies and participants. Improvements in participants’ perceptions of stroke recovery underscore the potential of EAT to enhance self-efficacy and quality of life post stroke. These findings align with the therapeutic goals of promoting neuroplasticity and regaining functional independence.

Systematic reviews have concluded that exercise in terms of targeting balance, core stability, reaching, weight-shift, gait training, or proprioceptive neuromuscular facilitation exercises could have significant positive effects in trunk control, sitting and standing balance, mobility, walking distance, and comfortable gait speed [65,66,67]. Common instruments, as in our study, were the BBS and TUG. In contrast to our study, there is one meta-analysis [66] that has shown that balance capacity improved in terms of the BBS with exercises targeting balance, weight-shifting, and/or gait training. No meta-analysis has been conducted in the literature specifically addressing stroke participants.

Overall, the literature highlights the positive effects of EAT on individuals with neuromotor, developmental and physical disabilities mostly in their balance, functional and motor ability and spasticity. Increasing self-competence for participation would further improve participants’ motivation for participating, leading to a better quality of life [45,49]. Quality of life is widely regarded as a multidimensional concept, encompassing physical, mental, and social well-being [8]. Our meta-analysis, including specific gait and balance ability (BBS/PBS, TUG, POMA), function ability (GMFM), and children’s quality of life (CHQ—Physical Domain), demonstrated significant differences in individuals with impairments who received EAT compared to those who did not, but only in Dimension E of the GMFM and TUG. EAT has the potential to enhance emotional, cognitive, and social well-being and positively influence social participation [8]. It is a viable intervention option for participants with cerebral palsy, multiple sclerosis, and stroke and for the elderly.

#### 4.1.5. Methodological Considerations

The methodological quality of the included studies varied, with randomized controlled trials generally scoring higher on the Downs and Black quality assessment tool. The substantial agreement between raters (kappa = 0.93) supports the reliability of the quality assessments. However, the low scores on certain items, such as the concealment of randomization and blinding, highlight areas for improvement in future research design. The lack of statistical significance in some meta-analyses could be attributed to high heterogeneity, small sample sizes, and variability in intervention protocols and outcome measures.

### 4.2. Clinical Implications

EAT offers a unique combination of physical, psychological, and social benefits, making it an effective, multidisciplinary intervention for individuals with disabilities. The findings support the inclusion of EAT in rehabilitation programs, particularly for populations such as children with CP, elderly individuals, and patients with MS or stroke. Healthcare providers should consider integrating EAT into patient care plans, emphasizing its potential to improve functional abilities and quality of life.

#### Study Limitations

Studies in the international literature regarding EAT show low evidence of effectiveness, low-quality studies, and inadequate sample sizes [8,68]. This review and meta-analysis integrated data from a diverse range of populations, providing a broad perspective on the benefits of EAT. The adherence to PRISMA guidelines and the use of robust statistical methods enhance the validity and reliability of the findings. However, it is important to consider some limitations when interpreting results of this systematic review and meta-analysis. Limitations include the exclusion of non-English studies, potential publication bias, and variability in the duration and intensity of EAT programs. Although there are many studies found in the literature, only a few could be meta-analyzed. So it was not possible to synthesize the studies and quantify the results. The fact that randomized and non-randomized studies were included in the meta-analysis may lead to errors in estimates. However, when only randomized sub-analysis was performed, estimates of statistically significant results practically did not change. The small number of studies examining each outcome in combination with the small number of participants in each study does not allow clear conclusions to be drawn. Finally, it should be noted that the literature search for this systematic review and meta-analysis was conducted on the 20th of April 2022, and, as such, studies published after that date are not included. While this may limit the inclusion of the most recent evidence, the review provides a comprehensive and methodologically robust synthesis of research conducted during a crucial period of development in the field of equine-assisted therapy for individuals with neurological, developmental, or physical disabilities. The findings offer valuable insights into the efficacy and applications of such interventions, as well as highlight important research gaps. Future reviews may extend this work by incorporating more recent studies to further examine ongoing trends and innovations in therapeutic approaches.

Further research is crucial to advance the understanding and application of EAT. Specifically, large-scale, multicenter randomized controlled trials are needed to strengthen the evidence base and provide more robust conclusions. Investigating the mechanisms underlying EAT’s therapeutic effects, such as its influence on neuroplasticity and muscle activation patterns, is essential to elucidate its physiological impact. Additionally, exploring the cost-effectiveness of EAT programs is necessary to promote broader implementation and accessibility. It is also important to examine the psychosocial and recreational benefits of EAT, which may enhance overall well-being and improve adherence to therapy. Finally, assessing the long-term sustainability of EAT benefits through extended follow-up periods will provide critical insights into its enduring efficacy.

## 5. Conclusions

This study confirms the multifaceted benefits of EAT for individuals with neuromotor, developmental, and physical disabilities. By improving physical functions such as balance and mobility and enhancing quality of life, EAT represents a valuable therapeutic modality. The integration of EAT into rehabilitation programs has the potential to improve outcomes and foster greater social participation and independence in affected individuals. Larger studies, preferably randomized, with better reporting for specific outcome tools should be conducted.

## Figures and Tables

**Table 2:** Results of meta-analysis.

	Number of Studies	I^2^	Heterogeneity P	Mean Difference (95% CI)	*p*
BBS (Elderly)	3	77.4%	0.012	0.11 (−1.46, 1.68)	0.889
BBS (Multiple sclerosis)	2	0.0%	0.559	2.48 (−1.79, 6.75)	0.255
BBS (Stroke)	2	0.0%	0.623	0.99 (−1.84, 3.83)	0.492
PBS (Cerebral palsy)	2	0.0%	0.629	3.21 (−1.82, 8.24)	0.210
**TUG (Overall)**	4	**0.0%**	**0.557**	**−0.61 (−1.05, −0.17)**	**0.006**
**TUG (Elderly)**	**3**	**0.0%**	**0.610**	**−0.60 (−1.04, −0.16)**	**0.007**
TUG (Stroke)	1	-	-	−5.30 (−14.13, 3.53)	0.239
GMFM 66	6	0.0%	0.771	0.67 (−0.31, 1.66)	0.182
GMFM 88	3	0.0%	0.650	2.92 (−1.19, 7.02)	0.164
GMFM A	2	0.0%	0.863	0.26 (−0.66, 1.19)	0.579
GMFM B	2	91.1%	0.001	9.79 (−12.28, 31.86)	0.385
GMFM C	2	70.5%	0.066	8.47 (−8.16, 25.11)	0.318
GMFM D	4	0.0%	0.749	0.11 (−0.99, 1.20)	0.847
**GMFM E**	**5**	**0.0%**	**0.951**	**2.48 (0.61, 4.34)**	**0.009**
POMA	2	0.0%	0.585	2.32 (−0.83, 5.46)	0.149
CHQ28 (physical domain)	2	0.0%	0.367	3.82 (−1.70, 9.34)	0.175

## Data Availability

The co-author can send all materials (data collection forms, data extracted from included studies, data used for all analyses, etc.) used for this meta-analysis.

## Abbreviations

The following abbreviations are used in this manuscript:

BBS	Berg Balance Scale
CHQ	Child Health Questionnaire
CP	Cerebral Palsy
EAT	Equine-Assisted Therapy
GMFM	Gross Motor Function Measure
MS	Multiple Sclerosis
PBS	Pediatric Balance Scale
POMA	Performance Oriented Mobility Assessment
TUG	Time up and Go test
